# Community Emergence of Cefixime-Resistant *Escherichia coli* Belonging to ST12 with Chromosomal AmpC Hyperproduction

**DOI:** 10.3390/antibiotics13030218

**Published:** 2024-02-27

**Authors:** Gloria Zaragoza, María Pérez-Vázquez, Laura Villar-Gómara, Andrea González-Prieto, Jesús Oteo-Iglesias, Juan-Ignacio Alós

**Affiliations:** 1 Servicio de Microbiología, Hospital Universitario de Getafe, 28905 Madrid, Spain; gloria.zaragoza.vargas@gmail.com; 2 Laboratorio de Resistencia a Antibióticos, Centro Nacional de Microbiología, Instituto de Salud Carlos III, 28222 Majadahonda, Spain; mperezv@isciii.es; 3 CIBER de Enfermedades Infecciosas (CIBERINFEC), Instituto de Salud Carlos III, 28029 Madrid, Spain; 4 Agencia Española de Medicamentos y Productos Sanitarios (AEMPS), Plan Nacional frente a la Resistencia a los Antibióticos (PRAN), 28022 Madrid, Spain; lvillar_externo@aemps.es; 5 Laboratorio central BRsalud, Hospital Infanta Sofía, San Sebastián de los Reyes, 28702 Madrid, Spain; agonzalez@ursalud.com

**Keywords:** *Escherichia coli*, ST12, AmpC, cefixime

## Abstract

*Escherichia coli* isolates that are resistant to cefixime and amoxicillin/clavulanic acid, but apparently susceptible to cefuroxime, with no ESBL identified, were initially detected in Madrid from urine samples in 2019. Throughout 2020 and 2021, all cases of community UTI by *E. coli* from six health areas in Madrid were studied. A representative sample of 23 cases was selected for further studies. The broth microdilution method and the agar diffusion method were performed to determine the antibiotic susceptibility. WGS was carried out for phylogeny, resistome and virulome analysis. Community consumption of third-generation oral cephalosporins in Madrid (2017–2021) was analyzed. A total of 582 (1.3%) *E. coli* isolates had the mentioned resistance profile. The mutation at position –32 (T > A) of the AmpC promoter was found in 21 isolates. No plasmid AmpC- or ESBL-encoding genes were detected. A cluster of 20 ST12 isolates was detected by cgMLST. A 6.2% increase in the consumption of third-generation oral cephalosporins, especially cefixime, was observed in Madrid. Chromosomal AmpC-hyperproducing ST12 *E. coli* isolates could be implicated in the increase in community UTI cases by cefixime-resistant isolates, which correlates with an increasing trend of cefixime consumption.

## 1. Introduction

Increasing resistance of Gram-negative bacteria to β-lactam antibiotics currently represents one of the main concerns worldwide. The primary mechanism of resistance is the production of β-lactamase enzymes, which have the ability to hydrolyze β-lactams.

*Escherichia coli* is an important etiological agent of both human nosocomial and community-acquired infections; in particular, *E. coli* is the main causative agent of urinary tract infections (UTIs) [1]. The acquisition of antibiotic resistance genes by *E. coli* makes the treatment of infections caused by these bacteria difficult. For example, extended-spectrum β-lactamases (ESBL)-producing *E. coli* isolates are resistant to many β-lactam antibiotics and usually co-producing mechanisms of resistance to other antibiotics [2].

Antibiotic-resistant *E. coli* can be selected in the human gut following the consumption of antibiotics and subsequently transmitted through the fecal–oral route. A previous study showed that 60% of community-acquired ESBL *E. coli* were attributable to human transmission [3].

A large proportion of UTIs are community acquired and are treated on an outpatient basis. Among the outpatient treatment options used are second-generation (cefuroxime) and third-generation (cefixime, cefpodoxime) oral cephalosporins.

Although ESBL are the most important oxyiminocephalosporin resistance mechanisms in *E. coli*, this resistance may also be mediated by AmpC β-lactamases [4]. Despite having been referenced under a different name in the 1940s, the first enzyme reported inactivating penicillin in *E. coli* was indeed an AmpC, which was before penicillin had been introduced in clinical use [5]. *E. coli* typically produce a class 1 cephalosporinase, encoded by the *amp*C gene, which is chromosomally located (c-AmpC). The term AmpC defines a class of enzymes that belong to the molecular class C according to Ambler’s structural classification of β-lactamases.

The expression of c-*amp*C in wild type *E. coli* isolates is low and not enough to confer clinically relevant resistance to beta-lactam antibiotics. Many mutations, insertions and gene duplication events have been shown to cause *c-ampC* hyperexpression, and this leads to varying spectra of β-lactam resistance dependent on the actual amount of AmpC produced. c-AmpC hyperproduction was first seen in *E. coli* from human clinical samples in 1979 and, for a period before the emergence of plasmid-mediated ESBLs, c-AmpC hyperproduction was the dominant mechanism of third-generation cephalosporin resistance in *E. coli* from humans [6]; however, this is no longer the case.

The AmpC phenotype may also result from acquisition of an AmpC gene encoded by a transferable plasmid (p-AmpC) [6]. The optimal treatment of infections caused by β-lactamase AmpC-producing bacteria depends on the early detection capacity of these enzymes.

In general, AmpCs exhibit a broad substrate specificity including penicillins (e.g., penicillin G; aminopenicillins such as amoxicillin and ampicillin; carboxypenicillins such as carbenicillin and ticarcillin; ureidopenicillin such as piperacillin), narrow-spectrum cephalosporins (e.g., cefazolin, and cefuroxime), oxyiminocephalosporins (e.g., cefotaxime, cefpodoxime, ceftazidime and ceftriaxone), cephamycins (e.g., cefoxitin and cefotetan) and aztreonam (variable), and their expression can confer resistance to all these compounds. The hydrolysis rate for fourth-generation cephalosporins (e.g., cefepime and cefpirome) is usually low, and that for carbapenems is very low, so that susceptibility to these drugs is usually maintained. Concerning inhibitors, AmpCs are usually resistant to β-lactam-based inhibitors (e.g., clavulanate, sulbactam and tazobactam) while being inhibited by the new non-β-lactam-based inhibitors (e.g., diazabicyclo-octanes, such as avibactam and relebactam, and boronates, such as vaborbactam) [7].

ESBL-producing strains usually have cefoxitin MIC ≤ 8 mg/L and elevated MIC to 3rd- or 4th-generation cephalosporins that recover with clavulanic acid, whereas AmpC-producing strains usually have cefoxitin MIC > 8 mg/L and elevated MICs to 3rd-generation cephalosporins (usually maintaining susceptibility to cefepime) whose activity does not recover with clavulanic acid.

In 2019, non-ESBL-producing *E. coli* isolates resistant to cefixime (MIC > 1 mg/L) and amoxicillin/clavulanic acid (MIC > 32/2 mg/L), and mostly with cefoxitin MIC ≤ 8 mg/L, began to be detected from urine samples in Madrid (Spain). This atypical profile caught our attention and, suspecting the emergence of a possible new plasmid AmpC production or unusual c-AmpC hyperproduction, we designed the present study.

The aim of this study was to determine the phenotypic and genotypic characterization, by complete genomic sequencing, of the community emergence of *E. coli* isolates with the aforementioned profile.

## 2. Results and Discussion

### 2.1. Patients and Isolates

Throughout the study period, 44,363 urine *E. coli* isolates from patients with community-acquired UTI were studied by the microbiology laboratories of the six health areas participating. Out of these isolates, 582 (1.3%) were resistant to cefixime and amoxicillin/clavulanic acid without ESBL production, which is a profile compatible with AmpC production; however, they showed cefoxitin MIC ≤ 8 mg/L or 16 mg/L.

The 23 representative isolates that were selected for further study were mainly from women (82.6%) between 18 and 65 years old (73.9%), and they were from all six participating health areas (range of isolates per health area: 2–9).

The prevalence of AmpC production in *E. coli* remains low in comparison with ESBLs. A study carried out with *E. coli* isolates resistant to amoxicillin/clavunanic acid showed an overall AmpC prevalence of 3.5% with approximately half of them being c-AmpC and the other half being p-AmpC [8]. The prevalence of c-AmpC in *E. coli* was 0.28% and 1.05% in other studies from Spain and Belgium, respectively [9,10]. Nonetheless, it is noteworthy to consider that the prevalence of 1.3% described in our study only refers to a specific profile of the overproducing c-AmpC with cefoxitin MIC ≤ 16 mg/L, so it is most likely undervalued.

### 2.2. Phylogenetic Analysis

**Figure 1:**
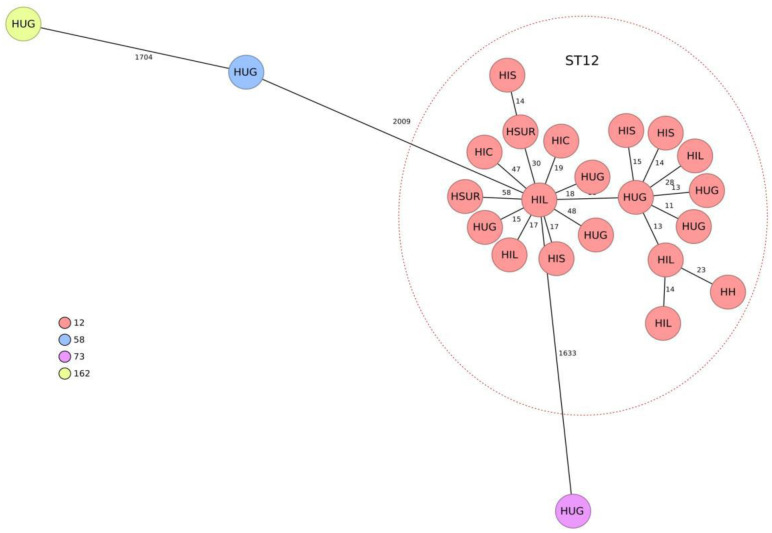
Population structure of the 23 representative cefixime-resistant *Escherichia coli* isolates studied. Distances shown in minimum-spanning tree are based on cgMLST of 2515 genes using the parameter ‘pairwise ignoring missing values.’ Colors in each circle indicate sequence type (ST) and the name as well as the health area of isolation. A red oval represents the ST12 cluster. HIL: health area of H. Infanta Leonor. HSUR: health area of H. del Sureste. HIC: health area of H. Infanta Cristina. HIS: health area of H. Infanta Sofía. HH: health area of H. de Henares. HUG: health area of H. Universitario Getafe.

The genomic assemblies of all 23 representative isolates, analyzed using the gene-to-gene approach and the allelic distance of cgMLST, are reflected in a minimum spanning tree (Figure 1). Twenty isolates were grouped into a main cluster belonging to ST12 according to the University of Warwick scheme. The average allelic distances between pairs of isolates in this cluster was 36 alleles (range 11–115). The other three isolates belonged to different MLSTs, according to the Warwick ST58, ST73 and ST162 (showing mean distances of 2405, 1940 and 2408 with the largest cluster, respectively). 

This study highlights the presence of genetically grouped ST12 isolates with the same resistance profile in community urinary tract infections from different areas of Madrid. Although ST12 has not been a particularly prevalent ST in *E. coli*, it is recognized as a cause of extraintestinal infections [11,12] and belongs to the usually virulent B2 phylogroup [12]. In a study carried out in Spain and France in 2016, 4% of 196 isolates causing urinary tract and other extraintestinal infections belonged to ST12 [11]. In a recent study, ST12 was isolated in 4.1% of UTI-causing strains in nursing homes, and it was significantly more frequent in recurrent UTI (9.3%) than in non-recurrent UTI (0%) [13]. Most of the information on the population structure of *E. coli* in Spain refers to multidrug-resistant isolates; ST131 has usually predominated in these populations, although in general, a great genetic diversity has been detected [12,14].

In contrast, we have not found reports of ST12 isolates with c-AmpC overproduction. In general, the *E. coli* population is genetically very diverse [15], and community dissemination of specific ExPEC clones has been rare and mainly associated with the successful B2/ST131 clone [16]. Historically, a high diversity of STs has been described in c-AmpC overproducers [10,17], which contrast with our results. In a study performed in Barcelona (Spain) on 240 *E. coli* with acquired or overproduced *bla*_AmpC_, none belonged to ST12 [9].

### 2.3. Resistome

Complete genomic sequencing analysis ruled out the presence of acquired genes encoding ESBL or AmpC-type plasmid β-lactamases in the 23 representative isolates studied.

Analysis of the c-AmpC gene promoter of *E. coli* showed the change at position −32 (T > A), located in box −35 (TTGACA), in 21 of the 23 studied isolates; all 20 ST12 isolates showed this modification. In the two remaining isolates (ST58 and ST162), two mutations were detected in the c-AmpC promoter: one at position −18 (G > A), located between boxes −35 (TTGACA) and −10 (TACAAT), and another at position −42 (C > T). 

Previous analysis of the promoter regions of c-AmpC *E. coli* genes confirmed the presence of two conserved regions, the −35 box and the −10 box, that are crucial for fixation of the σ subunit of RNA polymerase. The variation in these boxes, or in the distance between them, can condition the expression of the c-AmpC gene [18]. The −32 (T > A) modification has been previously described as one of the main modifications responsible for β-lactam resistance in *E. coli* by increasing AmpC transcription [18].

In addition, the resistome study in these isolates also demonstrated the presence of the genes encoding the β-lactamases TEM-1 (11/23, 47.8%), TEM-30 and TEM-34 (IRTs, one each, 8.7%). Other antibiotics resistance genes detected were *tet*B (tetracycline resistance; 14/23, 60.9%), *cat*B (chloramphenicol resistance; 1/23, 8.7%), *sul*1 and *sul*2 (sulfonamides resistance; 1/23, 8.7% of each) and *dfr*A12 (trimethoprim resistance; 1/23, 8.7%). Four (17.4%) isolates presented *gyr*A mutations related to resistance to quinolones.

### 2.4. Virulence Factors

Analysis of virulence genes showed a homogeneous profile in the 20 ST12 isolates including the adhesin coding genes *fim*H, *pap*C and *pap*A-F43 (20/20, 100%), *foc*C (19/20, 95%) and *sfa*D (18/20, 90%); however, another frequent adhesin gene in *E. coli* such as *afa* was not detected. ST12 isolates also had the toxin-encoding genes *vat*, *cnf*1 and *hly*A (100%) as well as the siderophore gene *iro*N (19/20, 95%). Other relevant virulence genes in *E. coli*, such as the *pic* and *sat* toxin genes, the siderophore gene *iut*A and the invasive protein gene *ibe*A, were not detected in the ST12 isolates of this study. All but one of the cefixime-resistant ST12 isolates had the capsular gene *kps*MIII_K96.

In this study, ST12/c-AmpC isolates show a high load of adhesin-coding genes that may favor adhesion to the urothelium. While the *fim*H gene, encoding type 1 fimbriae, is usually present in susceptible and antibiotic-resistant isolates of *E. coli* [11,12], the *pap*A, *pap*C, *foc*C and *sfa*D genes are not widely distributed. In a study involving 168 Spanish isolates susceptible (n = 56) and resistant (n = 112) to amoxicillin/clavulanate, these genes were generally detected in less than 50% of the isolates, and they were more frequent in susceptible strains (35.7%, 39.3%, 48.2% and 17.9%) than in resistant strains [12].

The presence of the *cnf*1 gene, encoding cytotoxic necrotizing factor 1, is also noteworthy, since it is an important virulence factor associated with some pathogenic strains of *E. coli* causing urinary tract infection and meningitis [19,20].

The presence of *iro*N, and other genes encoding siderophores, is one of the bacterial strategies to obtain iron from infected sites with very low iron availability [21].

In a recent study with Spanish and French *E. coli* isolates causing UTI and other extraintestinal infections, a strong correlation was observed between STs and virulence factors-encoding genes with a higher mean of virulence gene score observed in isolates belonging to ST12 and others STs belonging to the B2 phylogenetic group [11].

Contrasting with a virulent profile, as many isolates accumulated virulence factors, the prevalence of antimicrobial resistance was limited with no evidence of a multidrug-resistant emerging lineage, which was an occurrence in the ST141 clonal group [22].

### 2.5. Antimicrobial Susceptibility Testing 

In Microscan NM57 panels, all 23 isolates were resistant to cefixime (MIC > 1 mg/L) and amoxicillin–clavulanate (MIC > 32/2 mg/L). Susceptibility results to other β-lactam antibiotics included MIC ≤ 8 mg/L (susceptible or susceptible with increased exposure; 19/23 isolates) and MIC > 8 mg/L (resistant; 4/23) for cefuroxime; MIC ≤ 8 mg/L (19/23) and MIC = 16 mg/L (4/23) for cefoxitin; and MIC ≤ 1 mg/L (23/23; susceptible) for cefotaxime and ceftazidime.

The disk-diffusion method and the results of concentration gradient strips revealed resistance to cefixime (MIC: 16–64 mg/L; zone diameters: 6–12 mm), cefpodoxime (MIC: 8–32 mg/L; zone diameters: 6–13 mm), amoxicillin clavulanate (MIC > 256/2 mg/L; zone diameters: 6–15 mm) and cefuroxime (MIC: 16–64 mg/L; zone diameters: 11–18 mm). Cefoxitin MICs were between 16 and 64 mg/L with zone diameters of 6–18 mm. Both cefotaxime and ceftazidime MIC by concentration gradient strips were 1 mg/L in all 23 isolates.

Cefotaxime and ceftazidime activity did not increase with clavulanic acid (less than 5 mm difference in the inhibition zone diameter), ruling out ESBL production. However, there was a difference in inhibition zone diameter greater than 5 mm between ceftazidime, cefotaxime and cefixime with and without cloxacillin, indicating a possible production of AmpC.

In addition, most strains with this resistance phenotype, and all ST12 isolates, were susceptible to other non-β-lactam antibiotics (Table 1).

The differences in susceptibility to some β-lactam antibiotics depending on the method used are of concern. According to the phenotypic detection of c-AmpC and susceptibility results by concentration gradient strips and disk diffusion, we consider that there could be an underdetection of these strains in daily practice in microbiology laboratories using commercial Microscan microdilution panels. Usually, c-AmpC overproduction is suspected by the decrease in cefoxitin, amoxicillin/clavulanic acid and third-generation cephalosporins susceptibility, particularly ceftazidime; and by the lack of recovery of ceftazidime activity in the presence of clavulanic acid [23]. In this case, reduction in susceptibility to cefoxitin, cefotaxime or ceftazidime was not observed by microdilution panels, but it was detected by concentration gradient strips. 

Furthermore, in all isolates, resistance to cefuroxime was detected by the disk-diffusion method and concentration gradient strips, unlike the results obtained by Microscan microdilution panels in which 83% of the isolates were susceptible to this antibiotic. Cefuroxime is one of the outpatient treatment alternatives for UTI, which is why we should be especially careful with strains such as those detected in this study, which we believe should be reported as cefuroxime resistant. The marked susceptibility to the rest of the non-β-lactam antibiotics studied is noteworthy. Our most frequent lineage, ST12, has been previously associated with a low percentage of antibiotic multidrug resistance [11,24] which is consistent with our study, and it facilitates the outpatient treatment of infections caused by it.

However, and as generally considered, the emergence of community *E. coli* isolates resistant to cefixime is of concern, since cefixime is one of the few alternatives for the outpatient oral treatment of infections caused by multi-resistant strains.

### 2.6. Consumption of Cefixime and Other Third-Generation Oral Cephalosporins

The consumption of third-generation oral cephalosporins and, specifically, of cefixime in Madrid and Spain, is detailed in Table 2.

The use of 3rd generation oral cephalosporins decreased in Spain from 0.5453513 DIDs in 2017 to 0.5210577 in 2021 (−4.5% decrease; r^2^ = 0.26; *p* = 0.37) but increased in Madrid in the same period from 0.5925330 to 0.6292428 (6.2% increase; r^2^ = 0.61; *p* = 0.12). Cefixime consumption increased in Spain from 0.3008929 DID in 2017 to 0.3244793 DID in 2021 (7.8% increase; r^2^ = 0.04; *p* = 0.72), but the only statistically significant trend observed was the increase in the cefixime consumption in Madrid from 0.3589150 DID in 2017 to 0.4405656 DID in 2021 (22.7% increase; r^2^ = 0.95; *p* = 0.004).

The increase in the consumption of oral cephalosporins detected in this study, mainly cefixime and principally in Madrid, coincided with the emergence and spread of cefixime resistant non-ESBL *E. coli* isolates in UTI in this autonomous community. According to the preliminary molecular results detected in a small but representative group of isolates, which needs to be confirmed with subsequent studies, the cefixime consumption increase could be also related to the emergence of c-AmpC ST12 isolates in the community-acquired UTIs.

In a study in *E. coli* from dairy farms, an association between amoxicillin/clavulanate use and the risk of finding c-AmpC hyperproducers was found [25].

## 3. Materials and Methods

### 3.1. Isolates

From January 2020 to December 2021, 44,363 urine *E. coli* isolates producing community-acquired UTI from six health areas in the Community of Madrid (6,662,000 inhabitants) were studied for antibiotic susceptibility. Of them, 582 cases were resistant to cefixime and amoxicillin/clavulanate, had cefoxitin MIC ≤ 16 mg/L and did not produce ESBL; a representative sample of isolates from 23 different patients was selected for further phenotypic and genotypic analysis according to the following criteria: a single isolate per patient was included; at least two isolates per health area, one for each year of the study (2020–2021) were selected; and the number of isolates included per health area was proportional to the number of cases with the aforementioned profile that every area had (range of isolates per area: 2–9).

The *E. coli* identification was confirmed by MALDI-TOF mass spectrometry (MALDI Biotyper; Bruker Daltonics, Bemen, Germany)

### 3.2. Antibiotic Susceptibility Testing 

Primary susceptibility studies were routinely performed in clinical laboratories by broth microdilution by a semi-automated method (WalkAway, Beckman Coulter), mainly using Microscan NM57 panels, although cefoxitin susceptibility obtained with NUC95 and EN52 panels was also considered. EUCAST v12.0 clinical breakpoints and guidelines for *Enterobacterales* were used to interpret the data (http://www.eucast.org, accessed on 15 July 2023).

NM57 panels include cefotaxime/clavulanic acid and ceftazidime/clavulanic acid (both from 0.25/4 to 4/4 mg/L). These data allow interpreting a strain as ESBL positive if ≥8-fold reduction is observed in the MIC of any of the cephalosporins combined with clavulanic acid compared with the MIC of the cephalosporin alone; otherwise, the result is interpreted as negative.

In the representative sample of 23 isolates, the detection of AmpC β-lactamases was performed with cefotaxime, ceftazidime and cefixime disks with and without cloxacillin, and ESBL with cefotaxime and ceftazidime discs with and without clavulanic acid. 

In addition, susceptibility to different β-lactams (amoxicillin/clavulanic acid, cefuroxime, cefoxitin, cefixime and cefpodoxime) was determined by disk diffusion and concentration gradient strips methods and then interpreted according to EUCAST v12.0 breakpoints. For cefoxitin MIC, an EUCAST cut-off value of >8 mg/L was used as a highly sensitive indicator of AmpC-producing *Enterobacterales*.

### 3.3. Genomic Library Preparation, Sequence Analysis and Phylogenetic Analysis

All the 23 *E. coli* isolates were cultured on MacConkey agar (Becton Dickinson Microbiology Systems, Cockeysville, MD, USA) and incubated overnight at 37 °C. DNA was extracted using the QIAamp^®^ DNA Mini Kit (QIAGEN, Hilden, Germany) according to the manufacturer’s instructions.

All strains were subjected to WGS using the Illumina NextSeq 500 sequencer system (Illumina Inc., San Diego, CA, USA). A genomic DNA paired-end library was generated using the Nextera XT DNA sample preparation kit (Illumina Inc.). The quality of short reads was assessed using FASTQC, and they were assembled into contigs with Unicycler 0.4.8 [26]. The quality of the assembly was assessed with QUAST (http://quast.bioinf.spbau.ru/, accessed on 3 December 2022). Prokka v1.14-beta [27] was used for automatic de novo assembly annotation. Raw sequence data were submitted to the European Nucleotide Archive (PRJEB61431).

A core genome multilocus sequence typing (cgMLST) with 2528 genes provided by SeqSphere + 3.5.0 (Ridom, Münster, Germany) was applied to compare isolates.

Acquired antimicrobial resistance genes and chromosomal point mutations were analyzed using ResFindertool (CGE server: https://cge.cbs.dtu.dk, accessed on 5 September 2023) with an ID threshold of 98%, except for β-lactamase variants, which were determined with a 100% identity. 

In all 23 *E. coli* isolates, the presence of virulence-associated genes was identified using VirulenceFindertool (CGE server: https://cge.cbs.dtu.dk, accessed on 5 September 2023) with an ID threshold of 90%.

### 3.4. Antibiotic Consumption

The total consumption (hospital and community) of oral third-generation cephalosporins (cefixime, cefpodoxime, cefditoren and ceftibuten) in Madrid and Spain was determined according to the public and private health prescriptions for the period 2017–2021. Data were provided by the Spanish National Action Plan on Antimicrobial Resistance (PRAN) and coordinated by the Spanish Agency for Medicines and Medical Devices (AEMPS, Ministry of Health). They were obtained from the database of retail pharmacy sales from National Health System prescriptions and market research companies, covering nearly 100% of the Spanish population and both public and private prescriptions [28].

The information was tabulated, and the number of units was converted into defined daily doses (DDD) of active drug ingredients according to WHO methodology [28]. The number of DDD per 1000 inhabitants per day (DIDs) was calculated for each active drug ingredient. 

Trends in antibiotic consumption were examined by regression model analysis using GraphPad Prism software v.9.1 (GraphPad Software Inc., San Diego, CA, USA). 

## 4. Conclusions

An increase in cefixime resistance was detected in non-ESBL-producing urine *E. coli* isolates from Madrid. The preliminary molecular results obtained in a representative sample of isolates suggested that this increase could be at least partially related to the dispersion of overproducing c-AmpC ST12 isolates. 

Cefixime consumption significantly increased in Madrid from 2017 to 2021, which is a factor that could have exerted selective pressure for the selection of cefixime-resistant *E. coli* and, specifically, of ST12 resistant isolates.

The misidentification of cefoxitin susceptibility in theses isolates by some frequently used commercial panels, together with the slight affectation of cefotaxime and ceftazidime activity (remaining as susceptible according to EUCAST criteria), could lead to the under-diagnosis of these isolates.

*E. coli* is a good sentinel microorganism to detect evolutionary trends of antibiotic resistance in community-acquired infections. The design of comprehensive surveillance systems that include whole genome sequencing is necessary to confirm and trace the evolution of the trends such as those detected in this study.

## Figures and Tables

**Table 1:** Antibiotic susceptibility to non-β-lactam antibiotics according to EUCAST 2022 of the 23 representative isolates studied.

Non-β-Lactam Antibiotics	Susceptible (%)	Resistant (%)
Ciprofloxacin	22 (95.7)	1 (4.3)
Fosfomycin	23 (100)	0
Gentamicin	23 (100)	0
Tobramycin	23 (100)	0
Amikacin	23 (100)	0
Trimethoprim sulfamethoxazole	22 (95.7)	1 (4.3)

**Table 2:** Total consumption of oral third-generation cephalosporins (cefixime, ceftibuten, cefditoren and cefpodoxime) and cefixime specifically in Spain and Madrid.

Year	Oral 3rd Cephalosporins Spain *	Oral 3rd Cephalosporins Madrid *	Cefixime Spain *	Cefixime Madrid *
2017	0.5453513	0.5925330	0.3008929	0.3589150
2018	0.5672385	0.6184876	0.3169629	0.3809220
2019	0.5896015	0.6372055	0.3327773	0.3975886
2020	0.5041860	0.6365255	0.2929449	0.4044176
2021	0.5210577	0.6292428	0.3244793	0.4405656
r^2^/*p* **	0.26/037	0.61/0.12	0.04/0.72	0.95/0.004 ***

* Expressed in daily defined doses per 1000 inhabitants per day (DID). ** By simple linear regression model. *** Statistically significant trend.

## Data Availability

The datasets presented in this study can be found in the European Nucleotide Archive (PRJEB61431).
